# Always leave the audience wanting more:  An entertaining approach to stimulate engagement with health research among publics in coastal Kenya through ‘Magnet Theatre’

**DOI:** 10.12688/wellcomeopenres.16461.1

**Published:** 2021-01-11

**Authors:** Gladys Sanga, Irene Jao, Noni Mumba, Salim Mwalukore, Dorcas Kamuya, Alun Davies

**Affiliations:** 1 Health Systems and Research Ethics, KEMRI-Wellcome Trust Research Programme, Kilifi, Kenya, P.O. BOX 230-80108, Kenya; 2 Centre for Tropical Medicine and Global Health, Nuffield Department of Medicine, University of Oxford, Oxford, UK

**Keywords:** Magnet theatre, drama, engagement approach, public/community engagement, health research

## Abstract

**Background: **Magnet Theatre (MT), a form of participatory community theatre, is one of several public engagement approaches used to facilitate engagement between KEMRI-Wellcome Trust Research Programme (KWTRP) researchers and public audiences in Coastal Kenya. We describe how we used MT as an entertaining forum where audiences learn about research, and where researchers learn about how the public views research.

**Methods: **Drama scripts depicting community interaction with different aspects of research were developed iteratively with research staff, a theatre company and community members. Six fortnightly theatre outreaches per site over two months, attracting a total of 1454 audience members were held in Mida, a rural village 30 km north of Kilifi; and in Mtwapa, a peri-urban town 45 km to the south. Audiences were presented with dramatized health research-related dilemmas and subsequently invited to enact their responses. Evaluation comprised, notes and observations from meetings, rehearsals and outreaches, transcripts from a review workshop with repeat audience members (n=21), a reflection meeting with KWTRP engagement staff (n=12), and a group discussion with the theatre company (n=9). Discussions were recorded, transcribed, translated to English and analysed using thematic approach.

**Results: **Despite being costly in terms of time and expense, we argue that MT in public spaces can assist audience members to navigate ‘border-crossings’ between everyday contexts and scientific/research concepts. This can enable audiences to share their views and concerns and enact their responses to research-related dilemmas.

**Conclusions:** While reporting on MT’s successes, drawing from literature on rumours, we acknowledge the limitations of individual engagement activities in providing long-term solutions to address alternative interpretations and rumours about research, in the context of local and global inequities. MT, however, presents an opportunity for researchers to express respect to public audiences through making research more accessible and providing opportunities to listen to public views and concerns.

## Introduction

There is a growing body of literature emphasising the importance of engagement between researchers, community members and the broader public for a range of reasons; including raising awareness and understanding of research, decision-making about research participation, raising researcher awareness of public concerns about research and incorporating public views into research ^1– 3^. A range of engagement approaches have been described ^4^; however, descriptions of the use of Magnet Theatre (MT) as an approach for engaging the public with research are rare.

Theatre has been used for decades as a creative approach for fostering community engagement with sensitive, technical issues. According to practitioners, theatre helps to break down hierarchies for nurturing trust over time, allow different parties to share their perspectives in a safe space and facilitate cross-learning in an enjoyable way ^5^. Attitudes and behaviours are portrayed by antagonists and protagonists and explored jointly with participating audiences ^6^. Theatre has been used in different health fields including health promotion ^7^, health education ^8^, policy engagement ^6^ and to a lesser extent to engage the public with health research ^9, 10^.

For health promotion, community theatre performances in Bangladesh aimed at raising awareness of eclampsia and means of effective treatment ^8^. Though the evaluation only involved 15 audience participants, the authors present evidence that the initiative was well received and contributed to improved audience understanding related to eclampsia. In 2018, theatre was used for public engagement with antibiotic use in Myanmar ^11^. The authors report improvement in audience understanding of antibiotics and its use, and that audience preferred Forum Theatre as a form of engagement compared to conventional approaches to giving formal health talks.

While some articles convey the potential strengths of theatre as a means of health promotion and raising audience awareness of health issues, another use described in the literature is to engage audiences with health policy and health service implementation. For example, in Canada, theatre was used as a method of engaging public audiences in the development of health policy on preimplantation genetic diagnosis ^12^. The team drew on audience observation and post-performance discussions to gather key public views about a controversial genetic procedure for selecting the genetic characteristics of embryos created through *in vitro* fertilization.

In describing the use of theatre to engage audiences with ethical dimensions of healthcare, Bowman ^6^ reports that using theatre enabled audiences in London, UK, to understand and grapple with balancing the risks against the benefits related to critical healthcare decisions. She argues that using approaches such as theatre and radio, which are more familiar to members of the public than research settings, can contribute to reducing barriers to communication of complex research ethics topics, and increasing trust in healthcare.

‘Forum Theatre’ was used for faculty development at the University of Michigan’s (USA) multicultural medical school ^13^. The initiative involved intermittent pausing of the drama performance for audience reflection, problem analysis and facilitated discussion, and the process enabled audience insights to be incorporated into the way courses were implemented. The approach raised audience awareness of key planning and implementation issues, changed behaviour of the facilitators and offered strategies of addressing dynamics in the classroom ^13^.

The use of theatre in biomedical research over the last two decades is not widely described in the literature ^10^. A few articles, however, report success with the approach, for example, in South Africa, a study team used theatre to disseminate findings of research they conducted in six villages on understanding causes of stroke ^14^. Audiences in the six villages felt that portrayal of study findings was concurrent to real life happenings. During discussions, potential future research interventional thematic areas emerged.

In Cambodia, health research staff engaged with local communities towards developing a public theatre presentation aimed at educating communities about malaria transmission, prevention and treatment between health researchers and local communities ^15– 18^. This hybrid between engagement and health promotion involved working closely with 600 community members to develop a theatre production which reached audiences of 12,000 people across 20 villages. The authors present evidence that this outreach impacted audience members’ understanding of malaria and its prevention.

MT is a specific form of participatory community theatre that is conducted at an identified venue and at a specific time, over an extended period of time; aimed at “initiating and maintaining interactive communication with, and within, a community” ^5, 19, 20^. MT has a distinct component where the drama is paused at a point where the actors face a dilemma and the audience are invited to reflect on the problem, discuss possible solutions to the dilemmas and enact how the protagonists should proceed ^5^. This approach recognises that local socio-cultural norms and contexts influence individual responses to dilemmas and draw on these to elicit culturally appropriate solutions to specific challenges. In this way, the audience become actors taking responsibility for the solution. Following the performance, audience members reflect on the drama and apply the learning to their own lives to resolve challenges and dilemmas. Performing the drama at the same venue and time over a specified duration provides an opportunity for tracking attitude change over time and provides a forum for the magnification of behaviour change. The approach has been used for decades in grass-roots community HIV interventions across different countries to raise awareness, promote social and behaviour change and incorporate public views into planning and implementation of interventions and is identified as a one of the best practices in HIV prevention ^5, 20^. For example, the Program for Appropriate Technologies for Health (PATH) has used Magnet Theatre in Kenya since 2000 to encourage community communication, interaction and learning around HIV and AIDS issues, consequences of early marriage, and TB prevention and treatment ^20^. It has also been used to engage Zambian communities with malnutrition ^19^.

While there is a range of different uses of theatre in development, given its widespread use and success in health promotion and policy engagement, KWTRP engagement practitioners felt that MT potentially provided a locally appropriate and entertaining means of engaging public audiences with research. At the outset, it was felt that as in other settings, MT could raise public audiences’ understanding of research, nurture attitudinal change supportive of research and provide members of the public with fora to express their views and concerns in relation to research. There is, however, very little published literature describing the experiences of MT as a means of engaging the public with health research. This report focuses on Magnet Theatre as one of the public engagement approaches used at KWTRP and describes our experience of using the method.

## Methods

### Study site

KWTRP conducts integrated epidemiological, social, laboratory, clinical and health systems research, with results feeding into local and international health policies and practice. Research is supported by a Community and Public Engagement (CPE) strategy which includes several engagement approaches that have been implemented since 2006. KWTRP engagement is planned, implemented and evaluated by a multi-disciplinary team comprising the head and manager of engagement (NM and SM respectively), ethics and engagement researchers/evaluators (GS, IJ, DK and AD) and the Community Engagement (CE) staff. CE staff comprise a team of 15 trained and experienced male and female residents between the ages of 38 and 58 who come from Kilifi and speak the local languages fluently. For Kilifi residents, like other settings, health research is complex, filled with technical scientific jargon, and often not considered to be a priority topic for many. Magnet theatre was selected as one component of our broader CPE strategy to engage public audiences with health research in socially and culturally appropriate ways ^5^. The initiative, like KWTRPs other engagement approaches, aimed at nurturing co-learning and mutual understanding between researchers and publics and thus, enhancing the ethical conduct of research at KWTRP.

In March 2018, two areas 100 kilometres apart were selected for the MT initiative in Kilifi County. The first, Mtwapa, an urban town 40km south of Kilifi town, where minimal research has been done, and secondly, Mida, a rural area situated in the north where KWTRP census and research activities have been conducted for two decades. In keeping with the participatory nature of Magnet Theatre, site selection was done through a “walk-about” and consultation with village elders and chiefs at the two locations and members of Jukwaa Arts, the collaborating theatre company. Potential sites were considered in terms of their accessibility and capacity to safely host over 100 members of the public, their distance from potential distractions, and their being at a sufficient distance from quiet environments such as schools and hospitals to avoid disruption. The MT outreaches were held between March and September 2018 in Mtwapa and Mida respectively.

### 'Co-creating scripts

“Jukwaa Arts”, the local collaborating theatre company participated in a 3-day workshop at KWTRP to learn about health research and its social value, the range of on-going studies; and about research ethics. The workshop importantly, served as a platform for planning outreaches, discussing potential sites and introducing the research community to the theatre team and their work.

Theatre company members held informal talks with community members at the two sites to widen their understanding of community views about health research and inform themes and questions for the script content. From this, in consultation with engagement staff they drafted a script (see *Extended data* File 1 ^21^). For example, the first draft script for Mtwapa, entitled ‘ *Why do people refuse to participate in research*’, depicted a father who did not want his son to participate in research. Scripts were developed further and refined iteratively through discussion with the engagement team and during rehearsals. Scientific/research information was made as simple as possible, and technical jargon was avoided.

### Magnet theatre performances

Prior to the six fortnightly outreaches held at each site, crowds were sensitised through a combination of village elder communication, posters, a public-address system playing music and relaying messages, and a mobile music and dance group called ‘Simba Ropa’ ^1^. This approach attracted a crowd of about 70 people to each event, and this would increase as the play proceeded. Outreaches, run entirely by the Jukwaa Arts team and supported by KWTRP engagement staff, begun at 3.30pm and continued for 2 to 2 ½ hours.

Each MT performance involved several three-stage cycles. The first stage, acted by the theatre company for about 15 minutes, familiarised the audience with the drama’s characters and their lives, gave the audience an outline of the context, and arrived at a dilemma or tension point for the characters. The performance was then frozen for the second stage where the actors would freeze like statues providing an opportunity for audience discussion and debate. In the third stage members of the audience would take up the roles of some of the characters depicting how they would address the dilemma. This cycle was repeated two or three more times over about 35–45 minutes, enabling audience members to air their views and address any questions and concerns through dialogue. After the cycles, a Jukwaa Arts facilitator would wrap up the session and, on some occasions, invite subject experts from KWTRP to speak at the end of an outreach to address information gaps raised during the performances. At the end of the performance, the actors mingled further with the crowd while packing up thus providing further opportunities to respond to more questions, refer individual health concerns to appropriate health facilities and to bond with the audience.

Post-performance debrief meetings were held between KWTRP engagement staff and Jukwaa Arts team to explore what worked well and what needed to be improved in the next outreach.

### Monitoring and evaluation data collection

All monitoring and evaluation of this work was guided by a Theory of Change (see *Extended data* File 2 ^21^). Monitoring and evaluation methods aimed at assessing the influence and impact of the outreaches, and at guiding ongoing interventions, and comprised:

•   Minutes of planning meetings;
•   Field notes and structured observations of rehearsals and outreaches;
    ◦   12 drama performances’ monitoring reports (guided by an observation and reporting tool) (see *Extended data* File 3 ^21^);
    ◦   A report of a mid-term review meeting with Jukwaa Arts team and KWTRP engagement staff;
    ◦   Notes from post-performance de-brief meetings;
•   A final activity report from Jukwaa Arts;
•   A focus group discussion with 12 KWTRP engagement staff guided by a moderation tool (see *Extended data* File 4 ^21^);
•   A half-day reflective workshop with 21 repeat audience members (who consecutively attended four or more of the six outreaches) guided by a workshop moderation tool (see *Extended data* File 5 ^21^).
A monitoring tool was filled by a designated member of Jukwaa Arts during outreach, while the rest of the evaluation data was collected mainly by GS, NM, and IJ. KWTRP engagement staff and the evaluation team took field notes during all the rehearsals and outreaches and discussed their observations during post-meeting/performance de-brief meetings. Monitoring reports completed by the Jukwaa Arts team were verified by the KWTRP engagement staff and field-notes and notes from the de-brief meetings were added to complete the monitoring reports.

The reflective workshop with the repeat audience and the Jukwaa Arts team was a half-day activity conducted in December 2018, three months after the completion of all MT outreaches. Reflective workshop participants comprised audience members who were observed by Jukwaa Arts team to have attended four or more outreach seesions at either Mtwapa or Mida. Though challenging within a busy public context, the Jukwaa Arts team provided a list of outreach regular attendees. The evaluation team attempted to purposively select attendees of the reflective workshop from this list to ensure diversity in views based on gender, estimated age and religion. They were invited through letters followed up by phone calls a few days prior to the workshop. Out of the 30 repeat audience members invited (15 each from Mtwapa and Mida) 21 attended (six from Mtwapa and 15 from Mida). The reflective workshop was facilitated by the evaluation team at the KWTRP, during which audience members were divided into small groups for a facilitated discussion guided by a topic guide. Discussions were audio recorded and notes were taken by the evaluation team. Meetings were closed with a plenary session where the groups shared a summary of their views. This was important to enable participants to clarify and confirm aspects of the findings. In an attempt to reach data saturation from the repeat audience members, rather than facilitating several FGDs, we opted to invite as many repeat audience members as possible to one workshop in order to maximise the range of views.

An audio-recorded FGD was conducted with the KWTRP engagement staff in July 2019, which lasted 2 hours and 56 minutes with a short break in-between. During the FGD, engagement staff reflected on the summarised findings of the reflective workshop and shared their perspectives on the outreaches.

Audio recordings of the workshop discussions and the FGD were transcribed and Kiswahili content was translated to English. 

Data was organized in QSR NVivo 10 software and analysed using content analysis approach. An initial coding framework was developed by GS and IJ and reviewed by DK. The improved coding framework was shared and revised following inputs by SM, NM, and AD where any discrepancies were discussed and resolved. Finalization of the coding framework was done iteratively with the co-authors (see *Extended data* File 6 ^21^). Categories and themes were developed emerging from the data. A summary was then developed with the final themes derived from coding framework. The preliminary findings from the analysis of the FGD data was triangulated with the data from field notes and the reflective workshop. The findings were discussed in a meeting with KWTRP engagement staff for verification and comments which helped develop a final report.

Throughout the tool development, data collection and analysis, the researchers were aware of their positionality as KWTRP staff and how this could influence their personal views towards the evaluation of engagement activities. For this reason, researchers tried to be reflexive as much as possible ensuring they maintained neutrality during the interviews, carefully using probes and being conscious of and managing group dynamics during discussions; especially with their fellow staff; the KWTRP engagement staff and Jukwaa Arts team. Staff were encouraged to carefully consider any biases during post-meeting/performance de-brief meetings.

This evaluation work was reviewed and approved by the Kenya Medical Research Institute (KEMRI) Scientific and Ethics Review Unit (KEMRI/SERU/CGMR-C/075/3416). Written informed consent (see *Extended data* File 7 ^21^) was sought from repeat audience, Jukwaa Arts team and the KWTRP engagement staff who were involved in all interviews (focus group discussion, review meetings and discussions during the reflective workshop) and for audio-recording.

## Results

### Implementing MT

KWTRP staff were initially unfamiliar with magnet theatre and two aspects of the initial stages assisted in the smooth running of the programme: the three day training and community walks with KWTRP engagement staff and Jukwaa Arts team; and performing a play for the scientific staff in one of the weekly seminar. The former enabled scripts to be co-authored and objectives to be agreed upon, while the latter was aimed at mobilising staff to participate.

*“It was important for us to understand magnet theatre process so that when we are walking with them… we are able to support in meeting the objectives. For instance, this knowledge helped in the review of the script…”.* (P10, KWTRP engagement staff, Male)

Despite performing for the scientific staff, only two research staff attended the 12 community outreaches. While experienced community engagement staff were able to address most questions and issues raised, in some cases, the presence of experts would have been desirable.

Challenges were encountered in identifying suitable sites which were busy enough to attract a sizeable audience whilst not being too noisy for engagement and maintaining a reasonable distance from places which could not be disturbed, for example schools and offices. Specific challenges were raised by the sites’ proximity to busy roads, churches/mosques and informal bars selling palm wine, sometimes referred to as *‘mnazi dens’.*

*“So for me the Mida site was not very far from the road which… okay when a heavy machinery is passing attention of the audience; they looked at the road unlike for Mtwapa where it was in the heart of the village, although next to a mnazi den…”* (P10, KWTRP engagement staff, Male)

Despite site selection challenges, early evening outreaches were attended by a total of 1454 adults, including some children living around the identified venues and it appeared that the public promotion of the events were effective.

Ice-breakers generally put audiences at ease at the beginning of the outreach; however, KWTRP engagement staff observed that at the first two performances, despite repeated probing by facilitators, audiences were initially hesitant to share views and experiences and to contribute to discussions. KWTRP engagement staff felt that this hesitancy was alleviated, firstly through the professionalism, willingness to learn and respectful conduct of the Jukwaa Arts actors, secondly through the MT approach itself, and thirdly through the audience learning about the purpose of the outreach and the work of KWTRP. The following quotes illustrate this:

*“… we contracted a very experienced group and they really did a good job and they were the kind of people also who would receive feedback positively and improve whenever they are asked to”.* (P8, KWTRP engagement staff, Female)
*“The drama impressed people, and apart from being impressive, people got educated and got more informed”.* (Mtwapa Repeat Audience, Male)
*“…at our place there was a mosque…. When the time reached I would usually leave them to continue and I would go to the mosque to get prepared, but when the time to announce reached I would call the madam in charge "Madam stop the music we want to pray." And she would put the music off immediately I finished speaking. That is great respect.”* (Mida Repeat Audience, Male) **

It was felt widely that the crowd interaction spurred on by the ‘freeze’ point, an integral component of the MT approach, helped to nurture a rapport between the MT team and the audience. At intervals in the performance the facilitator halted the drama and opened the floor for discussion and invited two or three audience members to act out how they would respond to dilemmas. The growing audience discussion, reflection and laughter observed over subsequent performances evidenced this growing rapport.

### MT stimulating audience engagement with research

In all outreaches, audience members were given opportunities to ask questions and raise concerns about KWTRP’s research. The questions and concerns raised, spanned a wide range and comprised: that KWTRP promoted sex; questions about specific research procedures and blood drawing; the time taken from research conduct to getting results/findings (why some results are given immediately and others are not given); the difference between research and medical interventions (diagnosis and treatment); and aspects of research ethics, for example, KWTRP’s response in the event of research participant death or refusal and voluntary participation/participation in research.

*“What happens when one refuses to take part in research work, are there any consequences?”* (Mida Outreach Audience Member, Male)

Careful explanation of the purpose of the outreach was required to enable audience members to relax and participate actively in discussions. Jukwaa Arts actors observed that as audience members understanding of KWTRP grew, so did their willingness to participate in interactive components of the outreach. In Mtwapa for example, where research is limited to a KWTRP satellite conducting HIV research with key populations (men who have sex with men and sex workers), more general awareness raising of KWTRP’s work was required to create a rapport than in Mida where a considerably greater range of research and engagement takes place.

*“In Mtwapa … they started catching up after the first three performances because many had not participated in research. They used to think the many research studies involved only ‘homosexuals’, but there was so much going on [at KWTRP] rather than what they were thinking. So first we had to remove people from the misconception then bring them together, but in Mida they had done a lot of research and they had their questions ready. When you did something, they could see this had already happened; it was much easier but it’s a learning process, so it was worth it”.* (Jukwaa Arts Actor, Female)

Audience members suggested several ways to address perceptions of the KWTRP HIV Research clinic, for example, an attendee suggested that the clinic could be opened up to all community members for broader health services than just HIV. An attending HIV research staff member explained the research clinic’s remit, and this helped respond to some of the concerns raised about the clinic.

*“I personally I didn’t know about the Mtwapa [KWTRP]. I didn’t know what work they do, but one of them came and was asked what work they were doing there which he answered. So, I also understood that there is certain type of work that they are doing there”.* (Mtwapa Repeat Audience, Female) **

Some audience members felt that learning about KWTRP’s work enabled them to reconsider unsupportive rumours about the research institute and form their own views about research. For example, in some cases, audience members reassessed their prior view that the KWTRP HIV clinic promoted same sex relationships.

*“……When you meet with the [KWTRP] staff they will explain to you until you understand. So, with that we got some knowledge that [KWTRP] people are okay, they are not evil and all those people who speak have not had a chance to be informed, but whoever will be educated will understand”.* (Mida Repeat Audience, Male)

Gains in audience awareness of KWTRP’s main mandate in Mida, reported at the reflective workshop, appeared, for some, to alleviate fears they had about KWTRP. For example, a female repeat audience member shared that she used to run and hide when a KWTRP vehicle would be seen approaching her homestead. After attending the magnet theatre outreaches, her fears were allayed.

*“Initially before the Jukwaa Arts people came, whenever I saw the [KWTRP] staff I would run away or if I wouldn’t run away, I would hide [laughter]. I would instruct my child to say I am not at home if they asked for me. But this is because I hadn’t known who they [KWTRP] are. So, when they came to Mida with their play that’s when I got enlightened there. That’s when I came to know who [KWTRP] are and what they educate people on. That’s when I got the encouragement, right now if they come, I cannot run away from them”.* (Mida Repeat Audience, Female)

A frequently raised concern for Kilifi community members is why blood samples are drawn for research and specifically that the volumes drawn from small children are too large ^22, 23^. This concern was also raised during MT outreaches. In Mida, an invited research staff explained the difference between clinical and research procedures and demonstrated the blood volumes taken for research purposes using different sample collection tubes, syringes, and storage bottles containing medium (liquid into which a blood sample is drawn into). According to a repeat attender, this helped her to better understand why blood is drawn and what volumes are safe to draw ^24^.

*“Whenever they came to my place, when I saw them coming I would tell [the children] ‘Tell them mother is not around because I am not interested with their stuff, they are now coming for my child’s blood already,’ I would leave with the child. [Laughter] Do you hear that? All that was due to lack of knowledge but when I saw these plays and its information, I came to understand and now when they come, I wish they could take longer”*. (Mida Repeat Audience, Female) **

Another common concern raised by mainly women during community engagement activities, is that their husbands’ suspicion is raised when women receive out of pocket allowance for research participation. A male outreach audience member from Mida reported that attending an outreach helped him gain a better understanding about why payments are made when an individual participates in research.

*“All along, I have known them [KWTRP] to be paying people to participate in research. I accompanied my mother to [KWTRP] Kilifi, and I witnessed her being given a total of five hundred shillings for participating in research. I have now learnt that the money was not payment however it was fare reimbursement and compensation for her time she had spent during the research process….”.* (Mida Outreach Audience Member, Male) **

Whilst audience members’ narratives provide evidence of changes in attitude and reported behaviour, despite repeated engagement over the last decade or so in Kilifi, understanding of research, for example, the required blood volumes for research, reimbursement of participant travel costs and the distinction between research and diagnostic tests, remains challenging for many community members. This highlights the need for engagement to be on-going and sustained long-term.

### Overall learning for the KWTRP Team

From our experience, and from the testimonies of repeat attenders, it appeared that conveying research and KWTRP work through entertaining vignettes which were familiar to audiences, for example dramatized family tensions, supported the strengthening of the audience’s awareness and understanding of research and addressed concerns. It was also felt that that drama provided an entertaining way of reaching out to people with low literacy and limited access to information about health, health research and the work of KWTRP.

*“The drama had some parts which were interesting, and some were frustrating, even a [machete] was fished out to cut others, but there were certain learnings. That was not bad because if you were bad you would be helped to cross over to this side; that means you are at a better place…. It was play but it was amazing”.* (Mida Repeat Audience, Male)
*“Creating awareness through drama makes people understand more. Let’s say in the rural not all people have TVs to watch and be aware. Not many are literate. So, there’s that person who is learned who will see something in the newspaper and maybe he will understand, the one who neither has a TV nor a radio it becomes so hard for them. But if they go as a drama and create awareness to all people whether it will be about [KWTRP], HIV/AIDS and such things people understand more”.* (Mtwapa Repeat Audience, Female)

Performance observation notes provided evidence that outreaches were widely enjoyed and KWTRP facilitators felt that in contrast to large community meetings, the performances had combination of fun, seriousness and learning. Overall, facilitators recommended that magnet theatre be used alongside other engagement activities to reinforce learning. Concerns raised about research through the magnet theatre outreaches were similar to concerns raised through other KWTRP engagement approaches. That magnet theatre appeared to reach out to audiences other than those reached through the more conventional village meeting approach is an important learning. ********

Participating research staff reported that they gained an appreciation of community concerns related to their research work. Mtwapa HIV Research Clinic staff for example, reported that attending the outreach helped them understand the continued community concerns about research among Key Populations.

*“I was about to say there was active participation and it was an open forum where the community could easily vent out, the issues they had about KEMRI and its work, and at the end I think it cleared the perceived misconception which the community has about our work”.* (P5, KWTRP engagement staff, Female)

The CE team reported that magnet theatre allowed for a deeper interaction with the public compared to other engagement approaches, a sentiment which was echoed by the repeat audience.

*“I think one of the lesson that I learnt, through the magnet theatre is that having a repeated interaction with the community…brings the level of trust more to good levels that they can share deeper concerns which means maybe they could not have shared in just one interaction”.* (P4, KWTRP engagement staff, Female)
*“… by reading newspapers or road shows… people just pass, there is no contact for people to ask questions, for you to see and be able to ask questions. So, it will be better if [drama] will be used more and more”.* (Mtwapa Repeat Audience, Male)

Implementation, as well as being enjoyed by audiences, faced some challenges. The first was maintaining a consistent group of actors throughout the initiative. Some actors dropped out and were replaced over the duration of the project and this incurred additional time and costs in training new actors about KWTRP and its roles. Secondly, some engagement staff questioned the implementation cost of MT in comparison to other engagement activities. These, as well the cost of contracting an independent theatre company, include the time taken by KWTRP engagement staff to orientate the theatre company members to KWTRP’s work, to iteratively co-create a script and to attend rehearsals and performances.

*“…I feel maybe we need to think through the issue of cost…. in terms of resources; time, money and all that, and the outcome that we have or the people that we meet. For me I think it doesn’t really relate well because we reached very few people and used so much money that it would have been better if we even did an open day that would have brought in even better results than an MT”.* (P8, KWTRP engagement staff, Female)

Lastly, while community members reported that they learned from the dramas, some expressed a concern that the number of performances were insufficient. This suggests that careful consideration of the time and financial costs of MT are required for it to be employed as a long-term engagement approach.

*“…I thought they [drama outreaches] were continuing for me to learn more but it came to an end…”.* (Mida Repeat Audience, Female)

“Leaving the audience wanting more” is however a common sentiment in theatre which can also be interpreted as audience enjoyment, and creating a thirst for future engagement.

## Discussion

In our study we define a public space as a place that is accessible to all people and includes public squares, bus stops, roadsides and other places where people congregate and pass by. They range from quiet peaceful settings, to busy urban centres, abundant in disruption, noise and, of course, people. This contrast might precipitate an almost ‘no-win’ situation for engagement practitioners: a quiet site, while being ideal for performance and dialogue, may not attract large crowds, whilst a bustling noisy city square may attract a huge crowd but may be too noisy for meaningful engagement. To draw large public audiences, KWTRP engagement staff and Jukwaa Arts team opted for the latter, and drew from the experience and professionalism of the theatre company to address the consequences of their selection. Whilst practically challenging, a benefit of engagement in a public setting, according to some audience members, is that the public MT performances attracted audiences who would otherwise be missed by other common forms of engagement. That MT can potentially broaden audiences for engagement and convey science in familiar ways, enabling researchers to learn from wider publics is an important finding for public engagement with research.

Whilst distractions and noise are challenging, aside from the potential to draw large crowds, there are other benefits of engaging the public in their own environments. Several authors (see for example Bultitude and Sardo, and Gehrke) ^25, 26^ describe the desirability of engaging publics ‘organically’ *“in the places where they already exist and through those discourses and social practices by which they enact their status as publics”* ^26^. They argue that ‘organic approaches’ hand over control of the discussion to members of the public, enabling a more authentic sharing of views and perspectives. Though our evaluation is limited in its ability to explore the durability of gains in understanding over time, we feel that in the short term at least, magnet theatre enabled audiences to form opinions about research, based on a strengthened understanding, gained through watching and enacting familiar life vignettes in familiar spaces. In the field of science education, Aikenhead (1996) ^27^ likens the acquisition and understanding of scientific concepts, to crossing cultural borders from familiar ‘life-worlds’ to a largely unfamiliar ‘science-world’. Situating research and scientific concepts and processes within scenes and dilemmas familiar to audience members are likely to have eased ‘border-crossings’ into the world of science and to some extent enabled learning. Jegede, in his article on *the eco-cultural paradigm in science and mathematics education in Africa,* describes this type of learning as “ *a state in which the growth and development of an individual's perception of knowledge is drawn from the sociocultural environment in which the learner lives and operates”* (p. 124) ^28^. Thus, we hypothesize that the three elements of MT (acting by the theatre team, freeze point/debate and acting by the audience) assisted audience members to border-cross into the world of research enabling them to grasp unfamiliar concepts: first, in comparison to engagement activities held in research institutions, unfamiliar and potentially intimidating to non-researchers, situating engagement in familiar public locations is likely to have put audiences at ease with contributing their views and interacting; second, that vignettes presented during the plays closely resembled the everyday realities faced by people enabled them to relate to the characters and their contexts in relation to research; and third, those who enacted their responses to the dilemmas presented were able to incorporate their learning into practical actions, which is likely to affirm the learning.

Reflections of repeat attenders, and subsequent confirmation through community meetings suggested that acquisition of an understanding of KWTRP and its work may have addressed damaging rumours circulating in the community about KWTRP for some audience members. Geisler and Pool (2006) ^29^ argue that the origins of rumours about health research prevalent in sub-Saharan Africa and beyond, are likely to be more complex than just the result of knowledge deficits and that addressing knowledge gaps alone may at best only partially remedy the situation. They describe rumours as metaphors people use to express dissatisfaction and contempt towards inequities and wealth disparities between the global North and South, and between researchers, former colonial rulers and host communities. The periodic emergence of rumours surrounding research in Kenya, despite almost two decades of community and public engagement ^22– 24, 30– 32^, would confirm Geissler and Pool’s sentiment. We acknowledge that individual engagement initiatives may not provide ‘silver bullets’ to address community and public views influenced, largely, by considerable global and local inequities; however, they may address part of the problem and provide temporary respite through expressing respect to communities by listening to their views and concerns. While inequities are unlikely to be ameliorated in the short term, and that research requires public support, community and public engagement will need to be sustained over a long-term using a range of approaches to support the development and testing of new health interventions.

We have presented evidence that involving ‘expert researchers’ in MT performances supports learning for both audiences and participating researchers; however, persuading researchers to participate proved challenging. Whilst recognizing the busy schedules of researchers, we feel that the importance of involving them at the ‘frontline’ of engagement cannot be understated. Their presence not only adds credibility to the messages conveyed, but equally important, provides a demonstration of willingness to be part of a community. That MT was a novel and untested initiative for KWTRP may have resulted in a hesitancy on the part of researchers to participate. We hope that a documentation of the challenges and benefits of MT as a credible form of engagement through this publication, coupled with being able to see a video of the outreach, may convey the value of participation to researchers in future MT initiatives.

Implementing MT is expensive and time consuming. While it may be tempting to think that experienced engagement practitioners may be able to save on selection and contracting costs through implementing in-house MT initiatives, our experience was that the engagement benefitted considerably from the professionalism and specific skillset of the Jukwaa Arts team. Further, we interpret the audiences’ ‘wanting more’ as an indication of their enjoyment of the performances and a willingness to attend future MT initiatives. It’s likely that other forms of engagement, such as community meetings or open days could be more cost-effective, but whether they stimulate similar levels of empathy and enjoyment is unknown. Different engagement approaches have different purposes, and for this reason we recommend the consideration of using MT as a component of an engagement strategy, to stimulate enjoyment and co-participation in engagement.

## Conclusion

MT provided a means of nurturing public engagement with health research. Conveying research concepts through dramatized dilemmas, everyday issues, and moments of joy and conflict, provided an entertaining way for audiences to learn about research, and for researchers to learn about public questions and concerns. Though expensive in terms of time and money, we recommend MT as a complementary and enjoyable addition to research institutes’ engagement strategies.

## Data availability

### Underlying data

**Data available**: Data that may be made available include: data included in the manuscript in form of quotes; summaries of the main themes; and anonymized data transcripts of a half-day reflective workshop with repeat audience members, Jukwaa Arts team and group discussion with KWTRP engagement staff, in keeping with the conditions below.

**What uses are applicable**: As stipulated in the consent documents, data may be used to support public engagement by other researchers in Kenya or elsewhere, where the nature of the data might be considered relevant. For data not included in the manuscript, the consent form indicates that data sharing will require the approval of the KEMRI Wellcome Trust research Programme Data Governance Committee (see below).

**Conditions under which data will be available**: Data provided in the manuscript may be used without request but with reference to the full article including the data. Other data will be made available with the approval of the KEMRI-Wellcome Trust Research Programme Data Governance Committee (applications to Data_Governance_Committee@kemri-wellcome.org), only where anonymization can be adequately achieved to protect the privacy and confidentiality of the participants/respondents and any mentioned individuals and institutions, and where the proposed use is seen as relevant to the nature of the data. Where the DGC recommend this, the national KEMRI Science and Ethics Review Unit may also be asked to approve the proposed use. Conditions for data sharing are outlined in a KWTRP Data Sharing Agreement, including that:

— the requestor shall use the data only for the agreed purpose as stipulated in the application form and shall not use the data in such a way that causes damage or distress to the data subjects or communities involved in the research
— The requestor shall agree to at all times to keep the data strictly confidential, and ensure that the data users maintain confidentiality of the data
— The requestor shall not in any way attempt to seek to discover the identity of data subjects, to compromise or infringe on their privacy and confidentiality of their information.

### Extended data

Harvard Dataverse: Replication Data for: Always leave the audience wanting more: An entertaining approach to stimulate engagement with health research among publics in coastal Kenya through 'Magnet Theatre’. https://doi.org/10.7910/DVN/O9PNJY ^21^.

— Extended data File 1: Magnet Theatre drama script 1 (PDF).
— Extended data File 2: Theory of Change Magnet Theatre approach (PDF).
— Extended data File 3: Magnet Theatre Outreach Monitoring Tool (PDF).
— Extended data File 4: Magnet Theatre Reflective Workshop Tool (PDF).
— Extended data File 5: Interview guide KWTRP CE staff (PDF).
— Extended data File 6: Coding Framework developed during the analysis of the evaluation data (PDF).
— Extended data File 7: Informed Consent Form Magnet Theatre (English translation) (PDF).
— Extended data File 8: Authors’ Short Bio-sketches (PDF).

## Notes

^1^ *‘Simba Ropa’* is a local music group comprising two drummers, a trumpet player, and 2–3 singers. This troupe is very popular for mobilization and promotion purposes at the Kenyan Coast. They play their music while going around towns and villages attracting community members for activities of various kinds.
