# Supplementary material for: Always leave the audience wanting more:  An entertaining approach to stimulate engagement with health research among publics in coastal Kenya through ‘Magnet Theatre’
Source: Wellcome Open Res. 2021 Jul 2;6:2. Originally published 2021 Jan 11. [Version 2] doi: 10.12688/wellcomeopenres.16461.2 (PMC8008348; doi:10.12688/wellcomeopenres.16461.2)
Supplement: Peer review history

# Peer review: Reviewer response for version 2

**DOI:** 10.21956/wellcomeopenres.18781.r44761

**Published:** 2021-07-02

**Authors:** Nicola Desmond

**Affiliations:** 1 Liverpool School of Tropical Medicine, Liverpool, UK

This is a good revision - I have no further comments.

Is the work clearly and accurately presented and does it cite the current literature?

Yes

If applicable, is the statistical analysis and its interpretation appropriate?

Not applicable

Are all the source data underlying the results available to ensure full reproducibility?

Yes

Is the study design appropriate and is the work technically sound?

Yes

Are the conclusions drawn adequately supported by the results?

Yes

Are sufficient details of methods and analysis provided to allow replication by others?

Yes

Reviewer Expertise:

Medical anthropology, trust, risk, community engagement, ethics.

I confirm that I have read this submission and believe that I have an appropriate level of expertise to confirm that it is of an acceptable scientific standard.

# Peer review: Reviewer response for version 1

**DOI:** 10.21956/wellcomeopenres.18124.r42493

**Published:** 2021-03-29

**Authors:** Elizabeth Kimani-Murage, Hilda Owii, David Osogo

**Affiliations:** 1 Maternal and Child Wellbeing Unit, African Population and Health Research Center (APHRC), Nairobi, Kenya; 2 Maternal and Child Wellbeing Unit, African Population and Health Research Center, Nairobi, Kenya

**General Comments**

 This paper presents interesting and very useful information on how participatory visual methodologies approaches can be used to engage the public on research purpose and obtain the views of the public to inform research and the practice of research. The paper details how Magnet Theatre (MT), a form of participatory community theatre, was used to facilitate engagement of public audiences in Coastal Kenya by the KEMRI-Wellcome Trust Research Programme (KWTRP) researchers. The KWTRP used MT to help the audiences understand research and to help the researchers also understand the perceptions of the public regarding research. 

 KWTRP researchers worked with participatory community theatre artists, Jukwaa Artists, to engage the public with MT. A co-creation process between Jukwaa Artists and the research team, with the engagement of the local community took place to ensure that the Artists understood the research issues and the prevailing views in the community to help develop the engagement strategy.

 Several magnet theatre performances were conducted in public spaces in the community and attracted huge members of the public. Monitoring and evaluation was later done to evaluate the influences and impact of the magnet theatre outreaches.

 The results highlight how the participatory engagement helped the public understand research and dispel misconceptions regarding research that was conducted by KWTRP. It also helped the public to understand the conduct of research and why things happened the way they did. Drama helped the audiences, even those with low literacy to understand research and the purpose of research in an entertaining way.

 Participatory visual methodologies are increasingly a great way of engaging the public to help them understand research concepts and the conduct and purpose of research in a way that is most relevant to the public. These participatory methodologies help empower communities and augment their voices – even the most faint voices in the community because they are participatory and less technical. They help to bring the public at par with advances in science, thereby making research more relevant to the public. 

**Comments based on the review guidelines for methodological papers:**

*1. Is the rationale for developing the new method (or application) clearly explained?*

 Yes. The rationale for the use of magnet theatre to help engage the public in an entertaining way so as to help them understand research and also to help the researchers understand the views of the public is well explained. However, more details could be provided on the factors such as political, socioeconomic and cultural contexts/factors that informed the choice of using MT methodology as opposed to any other participatory methodology.

*2. Is the description of the method technically sound?*

 Yes. The process of us of magnet theatre to engage the public is adequately described and is technically sound. The description of the method described encompassed three main approaches to public engagement: public communication to transmit information on the topic at hand, public consultation to provide information through a co-creation process, and public participation where two-way interaction between ‘experts’ and the audience was promoted through the magnet theatre performances. Monitoring and evaluation methodology is also adequately described.

 However, given that that this was a participatory methodology approach, it would have helped for the team to provide some visual examples in the paper – in the methodology and in the results sections.

*3. Are sufficient details provided to allow replication of the method development and its use by others?*

 Generally details are given to enable replication. However, if possible, the authors could provide more information on:

 What a team should consider when planning and managing MT in public engagement.

 What other factors may have contributed to researchers’ hesitancy to participate. The one currently given is the fact that MT is a novel and untested initiative for KWTRP and could have contributed to the researchers’ hesitancy. Could other factors have come into play e.g. institutional constraints of time, support, resources and incentives? What lessons/recommendations to other similar projects to enable them ensure adequate involvement of researchers in public engagement activities?

 It would be helpful to have more elaboration on how co-creation occurred and the role of the public. Was there capacity building of the public on MT?

 It would be good to provide more information on whether there was an independent and active evaluation integrated into the design process to give additional reflection. This is because it seems that the team reflexivity, workshops and FGDs were only done towards the end of the process. Also, it would be good to know if the evaluation efficacy and efficiency of using MT? 

 The team mention that the approach is expensive in terms of time and expenses. More details on this would be useful. 

 More details on the intended and unintended impacts that MT had on the public, scientists, research institutions and for other actors would be helpful.

 Information on any expectations from the audiences and how these were managed would be helpful.

*4. If any results are presented, are all the source data underlying the results available to ensure full reproducibility?*

 The available related data is outlined. We believe the data will be adequately available to those who need to access it.

 The themes presented in the results section could be enhanced. For example, we could have themes around: how MT helped deal with misconceptions regarding research; experiences of the public with MT; experiences of the researchers with the engagement. 

*5. Are the conclusions about the method and its performance adequately supported by the findings presented in the article?*

 Yes to a greater extent the conclusions are supported by the data provided. However, this could include information on implications for research and practice. 

**Clarification queries**

 Did the repeat audiences represent particular population groups? How were they identified, invited and/or recruited?

 Related to the above comment on representation, were there any attempts to promote inclusion and diversity by involving marginalised and excluded groups as part of the repeat audiences? If so, how did the project reach out to them? If no, why not?

 What is the plan for uptake? Will wider uptake be promoted to contribute to wider attitude/mind-set change on research processes, beyond the groups that were convened? How will this be done? How will the feedback from the public be used at KWRTP?

**Grammatical/formatting edits**

 Need for consistency in use of abbreviation to depict magnet theatre (MT). There are several instances where the full words are used even after the abbreviation was given earlier.

Is the work clearly and accurately presented and does it cite the current literature?

Yes

If applicable, is the statistical analysis and its interpretation appropriate?

Not applicable

Are all the source data underlying the results available to ensure full reproducibility?

Yes

Is the study design appropriate and is the work technically sound?

Yes

Are the conclusions drawn adequately supported by the results?

Yes

Are sufficient details of methods and analysis provided to allow replication by others?

Yes

Reviewer Expertise:

Public Health; public health nutrition; public engagement.

We confirm that we have read this submission and believe that we have an appropriate level of expertise to confirm that it is of an acceptable scientific standard.

# Response

**Published:** 2021-06-22

**Authors:** GLADYS SANGA

**Affiliations:** KEMRI-WELLCOME TRUST RESEARCH PROGRAMME, Kenya

Many thanks for your review of our article, we respond to specific comments and suggestions you make below:

**Comment:** More details could be provided on the factors such as political, socioeconomic and cultural contexts/factors that informed the choice of using MT methodology as opposed to any other participatory methodology.

**Our response:** Thank you for your comment. We have added a paragraph on page 6 of the manuscript explaining what influenced the selection of MT.

**Comment:** Given that that this was a participatory methodology approach, it would have helped for the team to provide some visual examples in the paper – in the methodology and in the results sections.

**Our response:** Thank you for your comment. A link to the drama/video had been provided in methodology and discussion sections. ( https://vimeo.com/475818534)

**Comment:** What should a team consider when planning and managing MT in public engagement.

**Our response:** Thank you for your comment. The considerations have been discussed throughout the results, discussion and conclusion sections. They include the examples below:

- Considerations for selection of outreach sites that can attract a sizeable audience.
- Implementors should know the audience they are targeting.
- Involvement of research staff (not usually involved in engagement) for initial buy in and for support in implementation i.e. participation in field activities.
- Ensuring the outreaches are enough for audience to learn.
- Research institutions with limited budgets for engagement may be advised to initially prioritise other engagement methods in terms of, time and human resource.

**Comment:** What lessons/recommendations to other similar projects to enable them to ensure adequate involvement of researchers in public engagement activities? It would be helpful to have more elaboration on how co-creation occurred and the role of the public.

**Our response:** Thank you for your questions. We highlighted on page 16 the importance of involving researchers at the frontline in similar projects and had also explained about co-creation of scripts in the methodology section. We have added a statement on the same in the discussion section on page 16 of the manuscript.  

**Comment:** What other factors may have contributed to researchers’ hesitancy to participate. The one currently given is the fact that MT is a novel and untested initiative for KWTRP and could have contributed to the researchers’ hesitancy. Could other factors have come into play e.g. institutional constraints of time, support, resources and incentives?

**Our response:** We appreciate your comment. Other than the aspect of MT being a novel approach at the programme, we have highlighted on page 16 that MT is very time consuming; required travelling to sites far away from the centre, and therefore brought about constraints on researcher’s time to support this type of activity.

**Comment:** Was there capacity building of the public on MT?

**Our response:** Thank you for the comment. Capacity strengthening was not explicitly specified as an aim of this initiative and consequently we have not described it in the paper. However, we do acknowledge that capacity building of the public may have occurred in terms of strengthening their understanding of research and potentially their confidence to engage with research staff.

**Comment:** It would be good to provide more information on whether there was an independent and active evaluation integrated into the design process to give additional reflection. This is because it seems that the team reflexivity, workshops and FGDs were only done towards the end of the process. Also, it would be good to know if the evaluation efficacy and efficiency of using MT? 

**Our response:** Thank you for your comment. We have addressed this to some extent in the monitoring and evaluation data collection on page 8.

**Comment:** The team mention that the approach is expensive in terms of time and expenses. More details on this would be useful. 

**Our response:** We acknowledge your comment. In the text on pages 14 to 16 we have outlined ways in which this was time consuming for staff and we feel that since the aim of the evaluation was not to do an economic analysis or an expense analysis cost, we feel that this will give a different feel to the paper and this was more about evaluating whether it was practically doable, how it was experienced by people.

**Comment:** More details on the intended and unintended impacts that MT had on the public, scientists, research institutions and for other actors would be helpful.

**Our response:** Thank you for highlighting this aspect. In our findings section we have attempted to document all outcomes and influences of MT on audience views about research, both intended and unintended. For example, MT revealed public perceptions of the HIV clinic in Mtwapa, and how this changed as a result of the performances. While it was not entirely expected we didn’t present this as ‘unintended’ consequence, but we do present as an important finding within the text, both intended and unintended.

**Comment:** Information on any expectations from the audiences and how these were managed would be helpful.

**Our response:** Thank you for this comment. Audience expectations were met to some extent through initial discussions with community leaders about the themes for the dramas as described in page 4.  

**Comment:** The themes presented in the results section could be enhanced. For example, we could have themes around: how MT helped deal with misconceptions regarding research; experiences of the public with MT; experiences of the researchers with the engagement. 

**Our response:** Thank you for your suggestion. We made several attempts at laying the data out with the themes you presented but we found that our way provided the clearest framework for sharing our findings. 

**Comment:** The conclusions are supported by the data provided. However, this could include information on implications for research and practice. 

**Our response:** Thank you for this comment. In our conclusion we have emphasised the value of co-creation in the design and implementation of magnet theatre for engagement. We also recommend the use of MT to complement other engagement strategies.

**Comment:** Did the repeat audiences represent particular population groups? How were they identified, invited and/or recruited?

**Our response:** Thank you for this comment. We chose the MT approach in order to attract an urban population in Mtwapa and the more rural population in Mida. We didn’t specifically target any particular marginalized group, but considered the fact that poor urban people and poor rural people in Kenya are marginalized in the sense that they are not able to access or be exposed to research information and that’s why we went out to these specific places. This is explained in the 2 ^nd^ paragraph in the methods section on page 7.

**Comment: **Related to the above comment on representation, were there any attempts to promote inclusion and diversity by involving marginalised and excluded groups as part of the repeat audiences? If so, how did the project reach out to them? If no, why not?

**Response: **As is consistent with many community theatre approaches, the implementation team targeted public audiences who happened to be present in the vicinity of the site at the time. As such this was an organically diverse group spanning a rage of ethnic and religious groups, ages and gender.  A broad description of the audience composition has been given in the findings section.

**Comment:** What is the plan for uptake? Will wider uptake be promoted to contribute to wider attitude/mindset change on research processes, beyond the groups that were convened? How will this be done? How will the feedback from the public be used at KWRTP?

**Our response:** Thank you for this. We have described we had intended to keep MT as a component of engagement in the broader strategy, but we have not done so because of COVID-19 restrictions, as described in page 16.

**Comment:** There is a need for consistency in use of abbreviation to depict magnet theatre (MT).

**Our response:** Thank you for pointing this out. This was noted and the necessary edits were made throughout the manuscript.

# Peer review: Reviewer response for version 1

**DOI:** 10.21956/wellcomeopenres.18124.r42096

**Published:** 2021-02-01

**Authors:** Nicola Desmond

**Affiliations:** 1 Liverpool School of Tropical Medicine, Liverpool, UK

This paper reports on the use of Magnet Theatre as a form of community engagement in health research linked to the KEMRI-Wellcome Trust Research Programme in Kilifi, Kenya. The paper describes the development, process and evaluation of this community engagement strategy including consideration of the logistics that may enhance or impede engagement through this approach. The benefits of Magnet Theatre are clearly demonstrated and the challenges in implementing the approach considered. The paper focuses largely on the concept of this approach rather than on the actual impact of the approach in terms of increased knowledge and understanding of health research. Whilst it emphasises the overarching benefits of increased familiarisation and trust in health research conducted by the Research programme it does not attempt to specifically measure the extent of the influence of MT on research engagement. As such the paper is a useful and interesting introduction to the relevance of theatre as a tool for learning about research but does not provide evidence of its instrumental utility as a tool for increasing knowledge of research. There is emphasis on the cost (time and monetary) of implementing the approach which leads the reader to conclude that such an activity may be worthwhile in settings such as KWTRP defined by long-term research and therefore community engagement needs but perhaps less relevant in settings where there is less long-term commitment to the research population. I would suggest that the approach is highly suitable for programmes such as KWTRP, and indeed partners across the WT overseas Africa and Asia programmes but perhaps less so for other settings, unless the purpose of the MT itself is to engage communities about a specific research aim such as the examples cited in Canada, Bangladesh etc. This type of generic approach to familiarising communities to health research is, despite this, a valuable, entertaining and effective method.

 There are a couple of areas that could have been expanded in the paper, providing greater detail in order to promote the consideration of this approach amongst those who may be less convinced. Firstly the provision of a more structured evaluation to promote some measurement of spread including a breakdown of audience by sex and age, a longer term follow-up with community members as opposed to just those who were repeat audience members (by definition likely to be more engaged with the institution) to explore the sustainability of the method on future engagement specifically and the potential use of the MT method to 'spread' messages in the wider community (reflecting and potentially countering the spread of rumours in these settings). A more explicit comparison across the two sites might have been interesting especially given their descriptions as relatively more or less exposed to health research conducted by KWTRP. Whilst this was considered in the follow up evaluation (FGDs and workshop) it wasn't explicitly evaluated more formally.

 However, I acknowledge the challenges in conducting a more rigorous evaluation of the impact of MT and recognise that the authors did not set out to measure impact and the paper provides an interesting and highly relevant perspective on the use of MT as a general and effective engagement tool for increasing trust and familiarity with health research in a long-term research setting.

Is the work clearly and accurately presented and does it cite the current literature?

Yes

If applicable, is the statistical analysis and its interpretation appropriate?

Not applicable

Are all the source data underlying the results available to ensure full reproducibility?

Yes

Is the study design appropriate and is the work technically sound?

Yes

Are the conclusions drawn adequately supported by the results?

Yes

Are sufficient details of methods and analysis provided to allow replication by others?

Yes

Reviewer Expertise:

Medical anthropology, trust, risk, community engagement, ethics.

I confirm that I have read this submission and believe that I have an appropriate level of expertise to confirm that it is of an acceptable scientific standard.

# Response

**Published:** 2021-06-22

**Authors:** GLADYS SANGA

**Affiliations:** KEMRI-WELLCOME TRUST RESEARCH PROGRAMME, Kenya

Many thanks for your review of our article, we respond to specific comments and suggestions you make below:

**Comment:** The paper focuses largely on the concept of this approach rather than on the actual impact of the approach in terms of increased knowledge and understanding of health research. Whilst it emphasises the overarching benefits of increased familiarisation and trust in health research conducted by the Research programme it does not attempt to specifically measure the extent of the influence of MT on research engagement.

**Our response:** Thank you for this comment. We have clarified in the M&E section that the monitoring and evaluation methods aimed at documenting public views and concerns about research and assess its influence on public perceptions of KWTRP, as opposed to impact.

**Comment:** The paper does not provide evidence of MTs instrumental utility as a tool for increasing knowledge of research.

**Our response:** Thank you for this comment. As stated in the 1 ^st^ comment, the evaluation didn’t explicitly aim to measure quantifiable impact of MT on knowledge. We do provide qualitative evidence of participants learning about research and improving their perceptions towards research and KWTRP in page 6-7. In future evaluations we may explore the possibilities of systematic inquiry into knowledge increment.

**Comment:** There is emphasis on the cost (time and monetary) of implementing the approach which leads the reader to conclude that such an activity may be worthwhile in settings such as KWTRP defined by long-term research and therefore community engagement needs but perhaps less relevant in settings where there is less long-term commitment to the research population.

**Our response:** We have addressed this in the final paragraph of the discussion and agree with your comment that MT may only be applicable in some settings. We like this approach and we have amended the final paragraph of the discussion.

**Comment:** I would suggest that the approach is highly suitable for programmes such as KWTRP, and indeed partners across the Wellcome funded Africa and Asia programmes but perhaps less so for other settings, unless the purpose of the MT itself is to engage communities about a specific research aim such as the examples cited in Canada, Bangladesh etc. This type of generic approach to familiarising communities to health research is, despite this, a valuable, entertaining and effective method.

**Our response:** Thank you for your comment and for acknowledging the place of MT in engagement.

**Comment:** Firstly the provision of a more structured evaluation to promote some measurement of spread including a breakdown of audience by sex and age, a longer term follow-up with community members as opposed to just those who were repeat audience members (by definition likely to be more engaged with the institution) to explore the sustainability of the method on future engagement specifically and the potential use of the MT method to 'spread' messages in the wider community (reflecting and potentially countering the spread of rumours in these settings). A more explicit comparison across the two sites might have been interesting especially given their descriptions as relatively more or less exposed to health research conducted by KWTRP. Whilst this was considered in the follow up evaluation (FGDs and workshop) it wasn't explicitly evaluated more formally.

**Our response:** Thank you for this pertinent comment. As explained in previous responses the evaluation did not set out to formally compare the two sites. However, we do present evidence of the differences, for example, in Mtwapa where there is an HIV clinic which influenced community perceptions about KEMRI. We have added *Table 1* on page 10 showing the total attendance for all the outreaches in the two sites.
